# Honeybee Odometry: Performance in Varying Natural Terrain

**DOI:** 10.1371/journal.pbio.0020211

**Published:** 2004-07-13

**Authors:** Juergen Tautz, Shaowu Zhang, Johannes Spaethe, Axel Brockmann, Aung Si, Mandyam Srinivasan

**Affiliations:** **1**Beegroup Würzburg, Lehrstuhl für Verhaltensphysiologie und SoziobiologieWürzburg, Germany; **2**Centre for Visual Sciences, Research School of Biological Sciences, Australian National UniversityCanberra, Australian Capital TerritoryAustralia

## Abstract

Recent studies have shown that honeybees flying through short, narrow tunnels with visually textured walls perform waggle dances that indicate a much greater flight distance than that actually flown. These studies suggest that the bee's “odometer” is driven by the optic flow (image motion) that is experienced during flight. One might therefore expect that, when bees fly to a food source through a varying outdoor landscape, their waggle dances would depend upon the nature of the terrain experienced en route. We trained honeybees to visit feeders positioned along two routes, each 580 m long. One route was exclusively over land. The other was initially over land, then over water and, finally, again over land. Flight over water resulted in a significantly flatter slope of the waggle-duration versus distance regression, compared to flight over land. The mean visual contrast of the scenes was significantly greater over land than over water. The results reveal that, in outdoor flight, the honeybee's odometer does not run at a constant rate; rather, the rate depends upon the properties of the terrain. The bee's perception of distance flown is therefore not absolute, but scene-dependent. These findings raise important and interesting questions about how these animals navigate reliably.

## Abstract 2

After outdoor flight, honeybees represent distances they have travelled to find food differently, depending on the visual properties of the landscape

## Introduction

When a scout honeybee discovers an attractive patch of flowers, she returns to the hive and performs the famous “waggle dance” to advertise the location of the food source to her nestmates (von Frisch 1993). The dance consists of a series of alternating left-hand and right-hand loops, interspersed by a segment in which the bee waggles her abdomen from side to side. The duration of this “waggle phase” conveys to the potential recruits the distance of the food source from the hive: the longer the duration of the waggle, the greater the distance (von Frisch 1993). This information is used by the recruited bees to locate the food source. Clearly, then, the scout as well as the recruits are able to gauge how far they have flown in search of food. Early studies concluded that bees estimate distance flown by gauging the amount of energy they expend to reach the destination (von Frisch 1993; Neese 1988). More recent studies, however, are providing increasing evidence that this “energy hypothesis” is incorrect, at least for moderate distances of a few hundred meters (Esch et al. 1994, 2001; Esch and Burns 1995; Srinivasan et al. 1996, 1997, 2000). Over these distances, bees appear to gauge distance flown by measuring how much the image of the world appears to move in the eye en route to the food source (Esch and Burns 1995, 1996; Srinivasan et al. 1996, 1997, 2000; Esch et al. 2001). There are two kinds of experimental evidence that support this “optic flow” hypothesis. First, bees that fly a given distance close to the ground signal a much larger distance in their dances than do bees that fly the same distance at a considerable height above the ground and therefore experience a smaller rate of image motion (Esch and Burns 1996). Second, bees trained to fly to a feeder placed inside a short, narrow tunnel, the walls and floor of which are lined with a random visual texture, indicate a hugely exaggerated flight distance in their waggle dances (Srinivasan et al. 2000; Esch et al. 2001). Evidently, the proximity of the walls and floor of the tunnel greatly amplifies the magnitude of the optic flow that the bees experience, in comparison with the situation during outdoor flight in a natural environment. On the other hand, when the same tunnel was lined with axial stripes—so that a bee flying through it would experience very little optic flow because the stripes were parallel to the flight direction—the bees signaled a very small distance, even though they had flown the same physical distance as in the previous condition (Srinivasan et al. 2000). This experiment indicated that distance flown was being measured in terms of integrated optic flow, and not in terms of physical distance flown or energy consumed. If bees do indeed gauge distance traveled by measuring optic flow and integrating it over time, it is pertinent to enquire into the properties of their visually driven “odometer.” Given that the environment through which a bee flies can vary substantially in terms of its visual properties, such as color, contrast, texture, and the distribution of objects, it is important to know whether, and to what extent, the bee's perception of distance flown is affected by these environmental variables. In other words, how “robust” is the honeybee's odometer?

In a recent study, this question was explored by training bees to fly into a short, narrow tunnel (as described above), and analyzing the waggle dances of the returning bees as the texture and the contrast of the patterns lining the walls and the floor were systematically varied (Si et al. 2003). The patterns used were black-and-white stripes and sinusoidal gratings of various spatial frequencies and contrasts. This study revealed that the honeybee's odometer is indeed rather robust to changes in the visual environment. For a flight of a given distance into the tunnel, the odometric signal is relatively unaffected by changes in the spatial frequency or contrast of the gratings, as long as the contrast of the grating is above 20%. (Contrast is defined here as 100*(I_max_−I_min_)/(I_max_+I_min_),* where *I_max_* and *I_min_* are the intensities of the bright and the dark bars of the grating, respectively.) At contrasts below 20%, the strength of the odometric signal starts to decrease. Another recent study revealed that the visual odometer of the honeybee is “color-blind” and driven exclusively by the green receptor (Chittka and Tautz 2003).

Here we examine the robustness of the honeybee's odometer when it is performing in natural conditions, during flight through varying landscapes. Specifically, we compare the strength of the odometric signal during flight over land, where contrasts tend to be relatively high and textures are relatively rich, to that during flight over water, where contrast tends to be low and texture is sparse. The experiments described here involve training bees to fly over stretches of water or land, and comparing their waggle dances.

These experiments also provide us with the opportunity to address two further questions that are somewhat controversial and not yet completely resolved. One question relates to whether bees can, and do, fly safely over large bodies of water, such as lakes. Heran and Lindauer (1963), for instance, suggested that bees experience difficulty in flying across lakes, often losing altitude and plunging into the water. The other question relates to whether bees, having discovered an attractive food source positioned in the middle of a large expanse of water, can successfully recruit their nestmates, through their dances, to visit the food source (Gould and Gould 1988).

## Results

### Experiment 1

**Figure 1:**
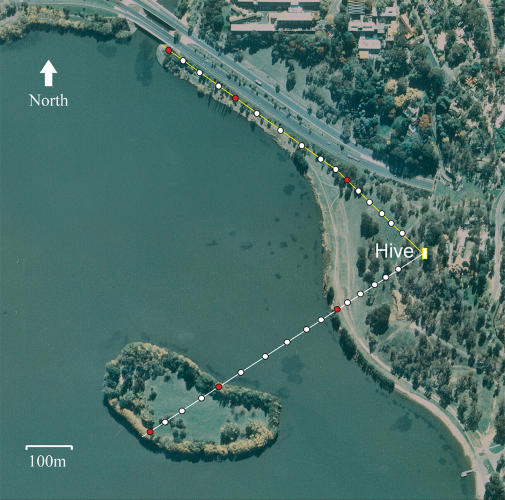
Aerial Photograph of Experimental Site In experiment 1 the bees were trained to fly due southwest from the hive initially over a stretch of land, then over water, and finally across the island. In experiment 2 the bees were trained to fly due northwest along a route that was entirely over land. The white dots depict successive locations of the feeding station. The red dots along the route of experiment 1 depict the shoreline stations, which represent the boundaries between segments 1 and 2, and between segments 2 and 3. The red dots along the route of experiment 2 represent the same distances from the hive as their counterparts in experiment 1.

In this experiment the feeder route was initially over land (segment 1), then over water (segment 2), and finally again over land (segment 3) (Figure 1). As the feeder was moved away from the hive in stepwise fashion, most of the marked bees followed. Virtually all of the marked bees continued to visit the feeder even when it was over water. Thus, the trained bees had no difficulty in flying over the water and finding the feeder in the boat.

The situation with unmarked honeybees recruited to the feeder by the marked scouts was rather different. A few recruits were observed at the feeder as long as the feeder was on land. Once the feeder was on the water, however, very few recruits were observed. Recruits reappeared when the boat reached the island. In fact, they were observed searching at the shore of the island shortly before the boat reached that point. Recruits were present at all feeder positions on the island.

**Figure 2:**
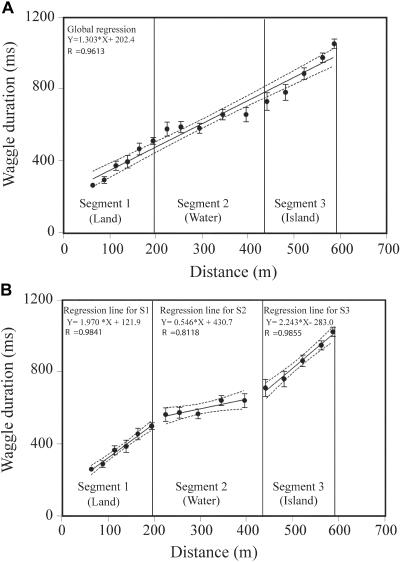
Variation of Waggle Duration with Feeder Distance in Experiment 1 In this experiment, flight was over land (segment 1), water (segment 2), and again over land (segment 3) (see Figure 1). Symbols depict mean waggle durations. Bars represent standard error of the mean. R, correlation coefficient. (A) shows a global linear approximation of the data, using a single regression for the entire data set (solid line). Broken curves depict 95% CIs for regression slope. (B) shows a piecewise linear approximation of the data, using separate regressions for the data over land, water, and the island. Equations represent regression lines.

For each feeder position along the route, the dances of the returning bees were filmed and analyzed as described in Materials and Methods. An example of a dance is given in Video S1. The variation of the mean waggle duration with feeder position is shown in Figure 2A. Waggle duration increased with feeder distance.

Figure 2A also shows the best-fitting straight line (a linear regression) through the data. The slope of this regression line is 1.303 ± 0.150, where the limits denote the 95% confidence interval (CI). The root mean square (rms) error between the data and this linear approximation is 42.3 msec.

Is this a good fit to the data? Visual inspection of the data in Figure 2 suggests that the rate at which the waggle duration increases with feeder distance (the slope of the curve) is higher in the first and third segments—where the bees fly over land—than in the second segment, where they fly over water. This impression is confirmed when linear regressions of the data are carried out separately for each of the three segments, as shown in Figure 2B. The results then reveal that the slope of the regression in segment 2 (water) is 0.546 ± 0.483, which is significantly lower than the slope in segment 1 (1.970 ± 0.348) and segment 3 (2.243 ± 0.500) (*p* < 0.02, pairwise comparison, Student *t*-test). On the other hand, there is no significant difference between the slopes for the land (segment 1) and the island (segment 3) segments (*p* > 0.2, Student *t*-test). Within each segment, the data fit a straight line quite well; the rms error between all of the data points and the piecewise linear approximation is 14.7 msec. This value is considerably lower than the rms error produced by the single straight line in Figure 2A (42.3 msec). Thus, the data are not well represented by a single linear approximation. They are better approximated by a piecewise linear relationship in which the slope over water is lower than that over land.

### Experiment 2

In experiment 2 the feeder route was of the same total length as in experiment 1, but was entirely over land (see Figure 1). In this experiment, unmarked recruits were observed at each feeder position.

**Figure 3:**
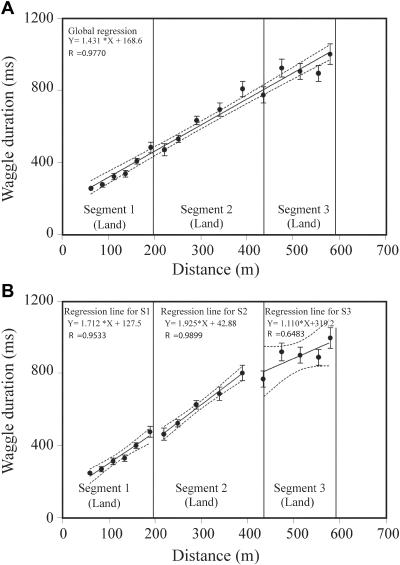
Variation of Waggle Duration with Feeder Distance in Experiment 2 For the purposes of comparative analysis, the data were artificially divided into segments corresponding to the land, water, and island segments of experiment 1 (see Figure 2). (A) shows a global linear approximation of the data, using a single regression for the entire data set. (B) shows a piecewise linear approximation of the data as they correspond to the segments of experiment 1. Other details are the same as in Figure 2.

The variation of the mean waggle duration with feeder position for this experiment is shown in Figure 3A. Here, again, waggle duration increased with feeder distance. The best-fitting straight line through the entire data set is shown in Figure 3A; the slope of the resulting regression line is 1.431 ± 0.695. The rms error between this line and the data is 37.9 msec. In addition, to enable a comparison of the data from experiment 2 with those from experiment 1, the same data set was artificially divided into three segments, of lengths 190 m, 240 m, and 150 m, as it was for experiment 1 (see Figure 2B), and a linear regression was performed separately on each segment. The results of this piecewise linear approximation are shown in Figure 3B. In this case, the slopes of the regression lines were 1.712 ± 0.526 (segment 1), 1.925 ± 0.358 (segment 2), and 1.110 ± 1.500 (segment 3). There was no significant difference between any of these three slopes (*p* > 0.10, pairwise comparison, Student *t*-test). The rms error between the piecewise linear approximation and the data was 41.6 msec, a value similar to that obtained with the single, best-fitting straight line (37.9 msec, Figure 3A). Thus, in this case, a piecewise linear approximation does not improve the fit. This analysis indicates that the data of experiment 2, in which the bees flew exclusively over land, is well approximated by a single line of constant slope. The slope of this best-fitting line is 1.431 ± 0.695, which is not significantly different from the slopes calculated for either of the land segments in Figure 2A (*p* > 0.05, Student *t*-test).

It is worth noting, however, that, in experiment 2, the three data points in the middle of segment 3 (Figure 3A and 3B) exhibit a much lower slope than the rest of the curve. A possible reason for this local variation will be discussed later.

We may summarize the findings of experiments 1 and 2 by saying that the mean waggle duration increases more rapidly with distance flown when bees fly over land, than when they fly over water. The slope of the mean waggle duration versus distance curve is three to four times greater over land. In other words, land provides a stronger odometric signal than does water.

Similar results were obtained when experiment 1 was repeated 3 weeks later using a different colony of bees. The new colony was placed in the same shed and allowed 10 d to acclimatize itself to the new location and familiarize itself with the surrounding terrain before the experiment commenced. The feeder was moved along exactly the same route as the original experiment 1. (Stakes had been placed on the ground to mark the locations of the feeding stations during the first experiment, to ensure that the same locations were used in the repetition. The feeder positions over the water were reproduced by using a marked rope, as described in Materials and Methods.) In this case, the slopes of the curve were 1.071 ± 0.127 (segment 1), 0.283 ± 0.693 (segment 2), and 1.990 ± 0.381 (segment 3). Here, again, the slope of the curve in segment 2 is substantially and significantly lower, as indicated by Student *t*-test results, than the slope in segment 1 (*p* < 0.01) and the slope in segment 3 (*p* < 0.001).

### Scene Analysis

**Figure 4:**
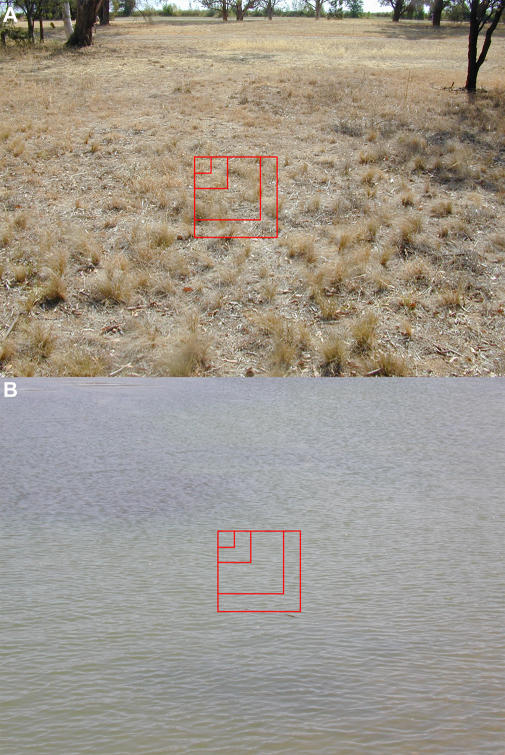
Land and Water Terrain Examples of scenes over land (A) and water (B) that were photographed and analyzed for mean contrast within windows of various sizes, as illustrated by the red boxes in the center of the photographs. Results are given in Tables 1 and 2.

As described in Materials and Methods, a digital camera was used to acquire samples of images of the visual scenes that the bees would have encountered during the flights over land and water. Two such samples, one over land and the other over water, are shown in Figure 4. The mean contrasts of these and a few other scenes, as measured within windows of various sizes (Figure 4) are given in Tables 1 and 2. In each case, contrasts were measured for images that were obtained with and without a green filter placed in front of the camera lens. The green filter was chosen to mimic the spectral sensitivity of the honeybee's green photoreceptor channel, the channel involved in the sensing of image motion (Lehrer 1987; Chittka and Tautz 2003). Contrasts tend to be slightly higher when scenes—over land as well as over water—are viewed through the green filter. It is clear from Tables 1 and 2, however, that, in general, contrasts tend to be substantially greater over land than over water, regardless of whether the scenes are viewed directly or through a green filter. The contrast over water tends to be lowest when there is no wind, i.e., when the water is still and there are no ripples. However, we did not investigate this latter phenomenon systematically.

**Table 1:**
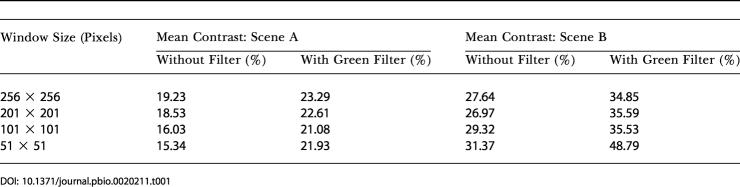
Mean Contrasts of Scenes over Land

**Table 2:**
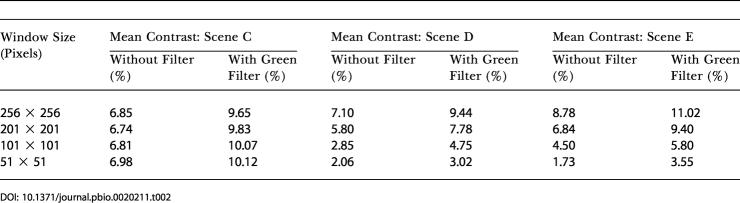
Mean Contrasts of Scenes over Water

We have assumed in this analysis that the image motion experienced by the bee is dominated by flow in the ventral visual field. This is not an unreasonable assumption, because the lateral structures (such as bushes and trees) were sparse and usually farther away than the ground, except in certain rare circumstances (see below).

## Discussion

Our results demonstrate, first of all, that bees can be trained to fly reliably and without accident over stretches of water that span a few hundred meters. This has also been reported in earlier studies (Heran and Lindauer 1963; von Frisch 1993; Gould and Gould 1988), although some studies (e.g., Heran and Lindauer 1963) mention that bees experience difficulty in flying across lakes, often losing altitude and plunging into the water.

As in earlier studies, we observed that individually marked bees trafficked regularly between the hive and the feeder, regardless of whether the feeder was on land or water. The short flight times (of a minute or two across the whole stretch of water, as established by radio communication) indicated that they had no difficulty in flying the direct route between origin and destination. We found, however, that the feeder attracted far fewer recruits when it was positioned over water. Although the scout bees that were trained to visit the feeder on land readily followed the feeder when it was moved over the water, recruits failed to appear once the water was reached. This was not because the trained scout bees ceased to dance once the feeder was over the water; they continued to dance with high vigor, and their waggles encoded a position over the water. Recruits reappeared when the feeder arrived at the island; in fact, they seemed to “anticipate” its arrival by patrolling the shore of the island even before the boat had landed there. Recruits were observed all along the route on the island, as well as along the entire stretch of the route used in experiment 2, which was exclusively over land.

One possible explanation for the lack of recruitment when the feeder was over water could be the relatively low slope of the distance indication curve in this region, which would lead to a less precise indication of the position of the feeder, making it harder to pinpoint. This explanation seems unlikely, however, since the boat in which the feeder was carried was large and conspicuous, and was the only object on the water.

Another possible reason for the lack of recruitment could have to do with the lack of assistance from experienced foragers. A recent study showed that experienced foragers can sometimes aid recruits in pinpointing a feeder by synchronizing their flights and performing buzzing flights around an unscented feeder (Tautz and Sandeman 2003). However, such assistance was not apparent in our study when the feeder was over the water. Why this assistance was not provided remains unexplained.

A third possibility is that, when inexperienced recruits fly over water, they fly at a higher or lower altitude than the trained scouts, and thus experience a different magnitude of optic flow. Consequently, they may search for the feeder at the wrong location. This possibility remains to be explored. What our observations do suggest, however, is that the *trained* bees fly at about the same height over land as they do over water (see below).

A fourth possibility relates to the controversial hypothesis, advanced by Gould and Gould (1988), that experienced bees have a “knowledge” of the surrounding landscape, including information about the existence and topography of bodies of water; and that they do not fly to locations signaled by other dancing bees that correspond to positions that are on the water, because such locations would be unlikely to bear food under natural circumstances.

The primary contribution of the present study, however, is the demonstration that the honeybee's odometer does not run at a constant rate in outdoor flight. The results shown in Figures 2 and 3 reveal that the mean waggle duration in the dance increases at a slower rate when bees fly over water than when they fly over land. In other words, for routes of the same length over land and water, the bees' perception of distance flown (as indicated by their dances) is smaller for flights over water. Thus, the honeybee's odometer runs at a slower pace when flight is over water.

There are a number of possible explanations for this finding. One reason could be that while flight over water is likely to stimulate only the ventral fields of view of the eyes with image motion, flight over land is likely to provide image motion signals in the lateral fields as well. In three earlier studies, honeybee odometry was investigated by training bees to fly through short, narrow tunnels lined with visual textures on the walls and/or or the floor. One of these studies (Srinivasan et al. 1997) suggested that the odometric signal is driven primarily by image motion in the lateral fields of view. However, the two others found that motion in the lateral as well as the ventral fields of view is important (Hrncir et al. 2003; Si et al. 2003). While the reasons for this discrepancy remain to be resolved, both studies suggest that lateral flow, if present, can contribute to the odometric signal. It must be noted, however, that in our experiments, the image motion experienced by the bee is likely to have been dominated by flow in the ventral visual field. This is because the lateral structures (such as bushes and trees) on both the water and land routes were sparse and were usually further away than the ground, except in rare circumstances.

A second reason for the different sensitivities of the odometer to flight over land and water might be that bees increase their altitude when flying over water, thus reducing the extent of image motion that a given forward motion of the bee would elicit in the eye. Although we do not have precise information on flight heights in this study, visual observation of bees approaching the feeder over the water suggested that they flew at heights of between 1 m and 2 m above the water surface, similar to the heights at which they are reported to cruise over land (Heran and Lindauer 1963). In fact, Heran and Lindauer (1963) reported that bees tend to fly lower over water, and seem to experience difficulty in maintaining the same altitude as they do over land. While we did not observe this phenomenon, their evidence as well as ours seems to argue against the possibility that the reduced sensitivity of the odometer over water is caused by flight at a higher altitude.

A third possible explanation has to do with the fact that the visual spatial texture of land could be considerably different from that of water. Investigation of this possibility would require a detailed analysis of the spatial frequency spectra of samples of land and water images, which we have not undertaken in the present study. However, a recent study examined the effects of varying visual texture on perceived distance flown in honeybees (Si et al. 2003). In that study, bees were trained to visit a feeder placed at the far end of a short, narrow tunnel, and their dances were analyzed as the texture and contrast of the patterns lining the floor and walls of the tunnel were systematically varied. The results revealed that perceived distance was almost invariant to changes in visual texture (i.e., changes in the spatial frequency content of the patterns).

The same study, however, also found that the odometric signal dropped substantially when the contrast of the pattern was reduced to a level below 20% (Si et al. 2003). This finding is consistent with our present field data, which suggest that the odometric signal is strong when the bees fly over land (which possesses a mean contrast of about 20%) but weak when they fly over water (which possesses a mean contrast of about 9%). Thus, a fourth explanation—and the most likely one—is that water surfaces exhibit a substantially lower visual contrast than do land surfaces.

Regardless of which of the above explanations is the valid one, our results indicate that the differences in the odometric signal between flight over land and over water are due to differences in the visual environment.

**Figure 5:**
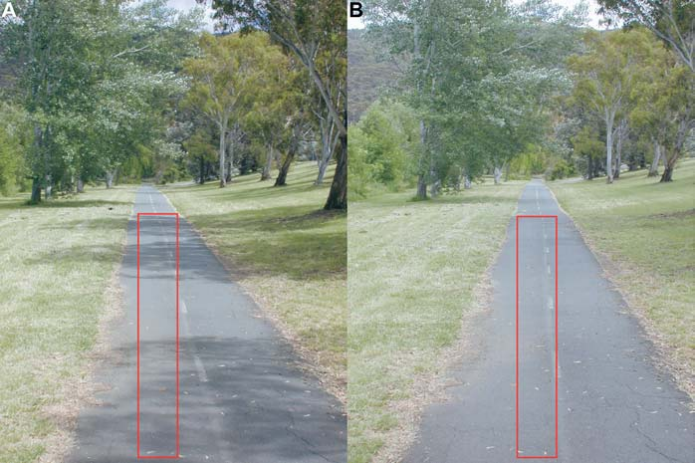
Terrain Along Which the Bees Were Trained to Fly in Segment 3 of Experiment 2 These two photographs show the terrain along which the bees were trained to fly in segment 3 of experiment 2. In (A) the sun was shining clearly, while in (B) it was behind a cloud. The rectangles depict the area within which mean contrast was measured (details in text).

Of course, the visual properties of land terrain can also vary considerably, depending upon the nature of the vegetation and on the existence of manmade structures. We suggest that this is the reason for some of the local fluctuations in slope that are evident in the data over land. In particular, we noted in the Results section that, in experiment 2, the three data points in the middle of segment 3 (Figures 3A and 3B) exhibit a much lower slope than the rest of the curve. This section of the land terrain was one in which the bees' flight took them along a paved bicycle path for a stretch of about 200 m. Two views of this section of the terrain, as would be experienced by a bee flying 1.7 m above the ground, are shown in Figure 5. These images were acquired without using any color filter (see Materials and Methods). It is evident that the surface of the bicycle path provides rather low contrast. (The mean contrast within the rectangle in Figure 5A was measured to be 14.30%.) The contrast of the surface is particularly low on a cloudy day (Figure 5B), when the surrounding vegetation does not cast any sharp shadows on the path. (The mean contrast within the rectangle in Figure 5B was measured to be 6.60%, which is even lower than that of most of the water surfaces that were measured.) The weather was indeed cloudy on the day the data for section 3 were obtained. This was confirmed by the weather entries in the experimental log book for that day, as well as by records from the Canberra Meteorological Station. Thus, the honeybee's odometer can run at different rates even on land, depending upon the nature of the local terrain. The three middle points in segment 3 show a progressively decreasing waggle duration, rather than a progressively increasing one. However, the decrease is not significant: A linear regression over these three data points reveals a slope of −0.369 ± 0.638, which is not significantly different from zero (*p* > 0.08). Thus, we interpret this as implying that the bees experienced very little optic flow during flight over this region. The final data point in section 3 shows an abrupt increase in waggle duration, compared to the previous three points. Interestingly, as we see from Figure 5, this is one of the rare segments of the bees' flight in which rows of trees appear close to the trajectory in the left and right lateral visual fields, potentially providing strong lateral flow.

It is instructive to compare our results with the findings from an earlier study by von Frisch and Lindauer (von Frisch 1993). In 1962–1963, von Frisch and Lindauer compared the dance tempos of bees returning from a 340-m flight over water with those of bees returning from a flight of the same distance over land (von Frisch 1993). (The “dance tempo” is the number of dance circuits completed in 15 s [von Frisch 1993].) They found no significant difference in the dance tempos under the two conditions. Based on this observation, von Frisch and Lindauer concluded that land and water drive the odometer at the same rate, and suggested, therefore, that the honeybee's odometer is driven largely by nonvisual signals. However, they did not measure the duration of the waggle phase, which is now considered to be the true representation of distance traveled (Seeley et al. 2000).

To investigate this issue more closely, we have measured four parameters of our dance data: mean circuit duration, mean waggle duration, mean return duration (the duration of the return phase of the circuit made during the dance), and mean dance tempo. The mean circuit duration is equal to the sum of the mean waggle duration and the mean return duration, and is proportional to the reciprocal of the dance tempo. The results of this analysis, for flights of the same distance over water and over land, are shown in Tables 3 and 4, respectively. These numbers were obtained by analyzing the dance data from experiments 1 and 2, respectively, for feeder distances ranging from 190 m to 390 m. Our results concur with those of von Frisch and Lindauer: As the feeder distance increased from 190 m to 390 m, the mean circuit duration exhibited a similar increase over land as it did over water (compare the first columns of Tables 3 and 4). Although the increase over land is somewhat larger than that over water, the difference is not statistically significant (*p* > 0.3, two-way ANOVA). The mean waggle duration increased much more rapidly on land than it does over water, just as Figures 2 and 3 indicate. However, the mean return duration increased much more rapidly on water than it did on land. As a consequence, the mean circuit duration showed a similar variation with distance over land as over water. This reconciles the present findings with those of von Frisch and Lindauer. The mean return duration is considered to be a measure of the “attractiveness” of the food source: the longer the duration, the lower the attractiveness (Seeley et al. 2000). Our data therefore suggest that (a) the attractiveness of a feeder diminishes as its distance from the hive is increased, and (b) the attractiveness decreases more rapidly with distance when flight is over water than when it is over land. The mean waggle duration, on the other hand, is a measure of the perceived distance flown; this quantity increases more rapidly on land than it does over water. Thus, in general, the odometer indeed runs at a faster rate over land than over water.

**Table 3:**
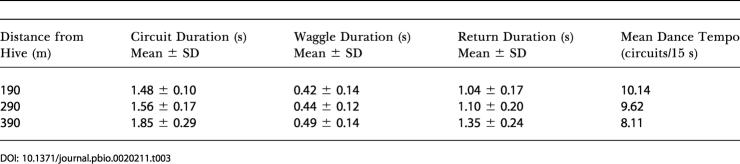
Analysis of Dances for Flights over Water (Experiment 1)

**Table 4:**
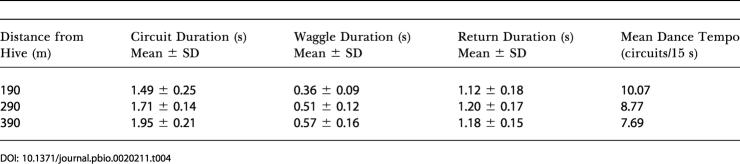
Analysis of Dances for Flights over Land (Experiment 2)

Our present findings confirm the suggestions from earlier work (Esch and Burns 1995, 1996; Srinivasan et al. 1996, 1997, 2000; Esch et al. 2001; Si et al. 2003) that the honeybee dance does not convey information about distance traveled with absolute accuracy. Rather, the distance that is indicated (measured in terms of the mean waggle duration) depends upon the optical environment in which the bee flies. The odometer runs faster in terrain that presents a high contrast and rich texture (such as land with dense vegetation) than in terrain that carries low contrast and sparse texture (such as a water surface). Although the visual movement detection system that drives the odometer is impressively robust to variations in visual texture and contrast—as revealed by an earlier study in which bees were trained to fly through tunnels in which the textures lining the walls and floor were systematically varied (Si et al. 2003)—this robustness is not perfect. The study by Si et al. (2003) indicated that, for a flight of a given distance, the odometric reading is largely independent of visual contrast, as long as the contrast is above 20%. Below this value, however, the odometric reading begins to decline. This critical value of contrast is in approximate agreement with the findings of the present study, where we have investigated the properties of the odometric signal in a natural, rather than an artificial, environment.

Given that the honeybee dance does not convey accurate information on distance flown, how do scout bees returning from a new food source usually manage to recruit other bees to visit it so quickly and so effectively? One explanation might be that the waggle dance conveys information on the direction as well as the distance of the food source. Thus, potential recruits that are persuaded by the scout's dance to seek out the source would fly in the direction signaled by the scout, and therefore experience approximately the same visual environment as the scout. Consequently, any terrain-induced variations in the odometric signal would be the same for the recruits as well as the scout, so that such variations would not compromise accurate pinpointing of the destination. The recruit would find the goal simply by flying in the specified direction until her odometric signal matched that indicated by the scout's dance. Thus, even though the scout's dance does not indicate distance in absolute terms, the recruits end up close to or at the correct location because their odometric signals evolve in the same way as that of the scout during the flight toward the food. Once the recruits are in the vicinity of the food source, the experienced foragers can assist them by providing visual and/or olfactory cues in the vicinity of the feeder to guide recruits to it (Tautz and Sandeman 2003).

## Materials and Methods

### 

#### Experimental site

The experiments were conducted on the shore of Lake Burley Griffin in Canberra. A two-frame observation hive was set up 190 m from the shore of the lake, with the entrance facing the shore (see Figure 1). The ground sloped gently downwards toward the shore. The terrain was grassy and interspersed with shrubs and eucalyptus trees. Beyond the lake shore, 240 m into the water, was an island (Springbank Island) measuring 150 m across along the line of sight from the hive. The island contained dense tree vegetation around its circumference, with grass and a few trees in the middle. Beyond the island was a further 1000-m stretch of water extending to the opposite shore of the lake.

#### Experiments

Two experiments were carried out, both using honeybees *(Apis mellifera)* from the same hive. In experiment 1, bees were trained to fly a route due southwest toward the island. This route comprised an initial stretch over land, followed by a stretch over water, and then again over land (across the island), as shown in Figure 1. The total length of this route was 580 m. In experiment 2, bees were trained to fly a route of the same total distance due northwest that was entirely over land (see Figure 1).

In experiment 1, about 20 bees were individually marked and trained to visit a feeder containing 1.0 M nonscented sucrose solution, initially placed 5 m from the hive. When the marked bees started to visit the feeder regularly, the feeder was moved step by step toward the shore. At each position, the dances of marked bees returning from the feeder were recorded at the hive. The distance from the hive to the lake shore was 190 m. The feeder was then placed in a rowboat and taken across the water, again stopping at several locations along the way to record dances back at the hive. In order to accustom the bees visually to the boat, we introduced them to the boat on land well before the water was reached. From about 50 m before the water's edge, the feeder was placed inside the boat. From this point on, the feeder was always in the boat, regardless of whether the location was on land or water. The stretch across the water was 240 m long. Upon reaching the shore of the island, the feeder (still placed in the boat) was moved stepwise across the island to the opposite shore, again recording the dances of bees returning from each location. The stretch across the island was 150 m long.

The boat was an inflatable dinghy capable of carrying four adults in addition to the feeder. It was colored bright yellow to facilitate visual detection from a distance by the bees. A view of the boat on the lake is given in Figure S1. A buoyant rope, carrying brightly colored markers at 10 m intervals, was used to measure out distances on land as well as water. During periods of strong wind or water currents, a stable position over water was maintained with the aid of an anchor, supplemented when necessary by compensatory paddling.

The weather was sunny and calm through most of the study, except for a few days (see below). The wind speed rarely exceeded 20 km/h. Most of the time, there were only a few ripples on the water surface. Temperature and weather conditions were recorded through the course of the experiment and were supplemented by records from the Canberra Meteorological Station.

There were always at least two experimenters at the feeder (regardless of whether the feeder was on land or water), and two experimenters at the hive. The visit of each marked bee at the feeder, and its subsequent dance in the hive, were followed through radio communication between the feeder and the hive. At each feeder position, recording of a given bee's dances was commenced after it had made three visits to the feeder. This was done to allow adequate time for the dances to adjust to each new feeder position. Data were collected from between seven and 14 different individually marked bees at each feeder position. This procedure required a stay of about 30–60 min at each feeder position.

In experiment 2, about 20 bees were trained by moving a feeder step by step from the hive, as in experiment 1, but along a route that was entirely over land, as described above. The total length of this route (580 m) was identical to that in experiment 1, thus enabling a direct comparison of the of the bees' dances along the two routes.

#### Recording and analysis of bee dances

The observation hive was housed in a specially constructed shed that afforded a weatherproof environment for observing and filming bee dances. Dances of marked bees were filmed at 25 frames/s, using a Sony (Tokyo, Japan) DCR-TRV310E video camera mounted on a tripod placed adjacent to one face of the observation hive, near the entrance. A mechanical gate at the entrance to the hive ensured that bees entered (and left) the hive only on the side facing the camera, thus facilitating the filming of dances.

Bee dances were analyzed frame by frame to measure the mean duration of the waggle phase. The waggle duration was considered to be a measure of the bees' perception of the distance flown from the hive to the feeder: the longer the waggle duration, the greater the perceived distance (Esch et al. 2001). A total of between 68 and 217 dances were analyzed for each feeder position, from between seven and 14 individually marked bees. A total of over 6,000 dances were evaluated in the study.

The dances were evaluated as follows. For each dance, the mean waggle duration was estimated by averaging the waggle durations over all loops. Then, the mean waggle duration for each bee was obtained by averaging the mean waggle durations over all of its dances at that feeder position. Finally, the mean waggle duration of all bees was calculated from the mean waggle durations for the individuals. The standard error of the mean was also calculated and displayed in the graphs (see Figures 2 and 3). Linear regressions of the data, and 95% CIs for the slopes of the fitted regression lines, were computed using the GraphPad Prism (GraphPad Software, San Diego, California, United States) statistical analysis package. Regression slopes of different data sets were also compared using the same package, which implemented the slope comparison test described in Sokal and Rohlf (1995).

#### Scene photography and analysis

Samples of the visual scenes that the bees would have experienced while flying over land and water were acquired by digital camera (Coolpix 950, Nikon, Tokyo, Japan), which produced color images of 1200 × 1600 pixel resolution. Sections of these images were analyzed to compare the mean visual contrast over land with that over water. The intensity of the image at each pixel was taken to be the average of the values of the three color subpixels at that location. These images were taken on a calm day with weather conditions similar to those on which the experiments were conducted. The surface of the lake was smooth, with relatively few ripples.

The mean contrast in an image section was computed as the ratio of the standard deviation to the mean value of the intensities of all the pixels within that section, and was expressed as a percentage. It was recently demonstrated that the odometer is “color-blind” and is driven primarily by the green receptor channel (Chittka and Tautz 2003), as are other motion-sensitive pathways in the bee (Lehrer et al. 1988; Zhang et al. 1990; Zhang and Srinivasan 1993). Therefore, each scene was photographed twice: once without any color filter, and once through a color filter with a spectral transmission that approximated the spectral sensitivity of the honeybee's green receptor (B+W 30061 3X MRC [Schneider-Kreuznach, Bad Kreuznach, Germany]; with peak transmission at 530 nm and a bandwidth of 120 nm at half sensitivity).

## Supporting Information

Figure S1 View of Boat at One of the Training Positions on the Lake (1.8 MB JPG).

Video S1 Marked Bee Dancing Upon Return from the Feeder When the Feeder Is Positioned 60 m into the Lake (250 m from the Hive) (27.8 MB AVI).

## Abbreviations

CI: confidence interval
rms: root mean square
SD: standard deviation
