# Supplementary figures and images for: Honeybee Odometry: Performance in Varying Natural Terrain
Source: PLoS Biol. 2004 Jul 13;2(7):e211. doi: 10.1371/journal.pbio.0020211 (PMC449896; doi:10.1371/journal.pbio.0020211)

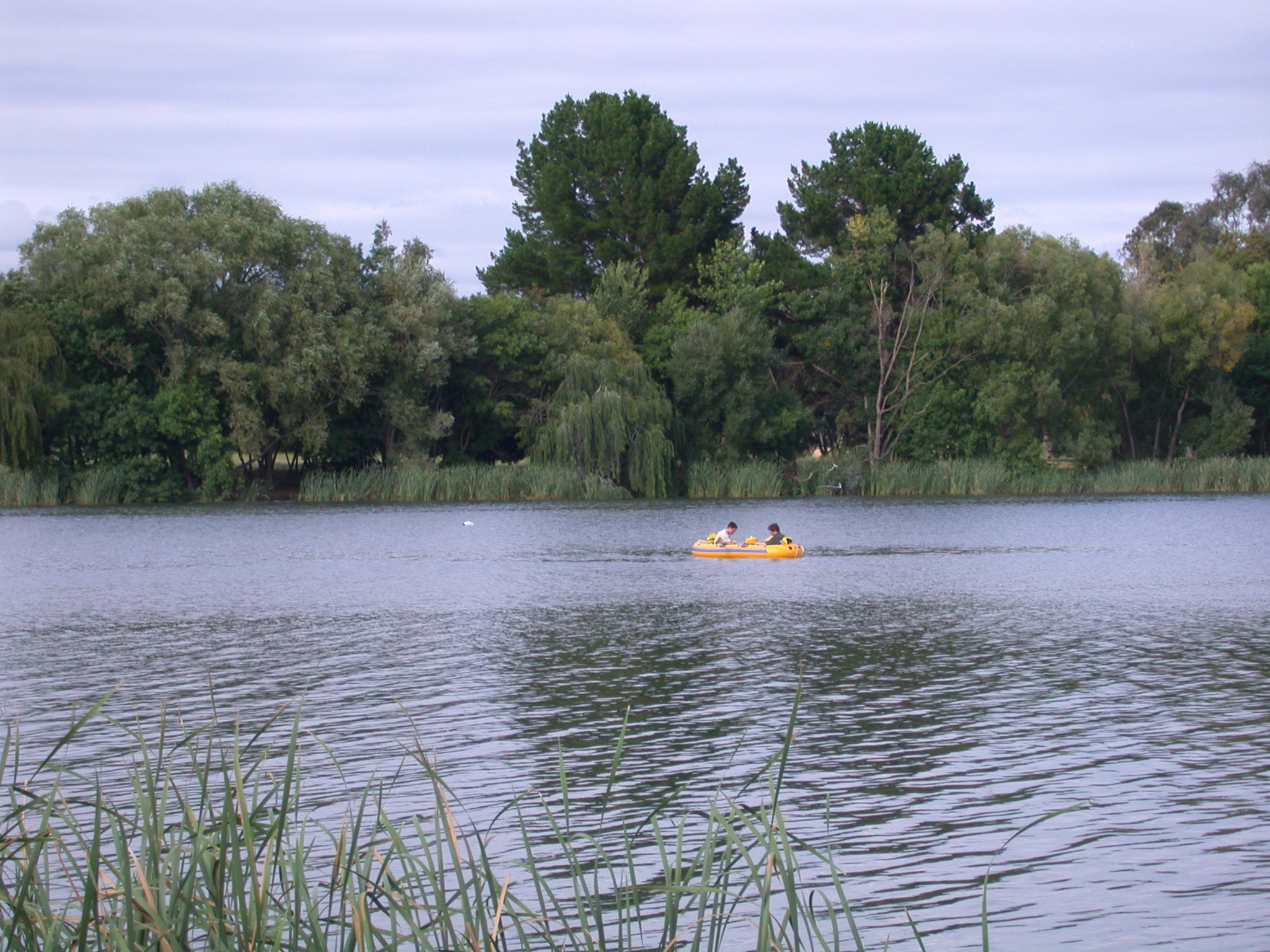

Supplement: Figure S1 — (1.8 MB JPG). [file pbio.0020211.sg001.jpg]
